# Different BCS Class II Drug-Gelucire Solid Dispersions Prepared by Spray Congealing: Evaluation of Solid State Properties and In Vitro Performances

**DOI:** 10.3390/pharmaceutics12060548

**Published:** 2020-06-12

**Authors:** Serena Bertoni, Beatrice Albertini, Nadia Passerini

**Affiliations:** Department of Pharmacy and BioTechnology, Alma Mater Studiorum-University of Bologna, Via S. Donato 19/2, 40127 Bologna, Italy; serena.bertoni4@unibo.it (S.B.); beatrice.albertini@unibo.it (B.A.)

**Keywords:** microparticles, spray chilling, spray cooling, Gelucire, semicrystalline solid dispersions, carbamazepine, tolbutamide, cinnarizine

## Abstract

Delivery of poorly water soluble active pharmaceutical ingredients (APIs) by semi-crystalline solid dispersions prepared by spray congealing in form of microparticles (MPs) is an emerging method to increase their oral bioavailability. In this study, solid dispersions based on hydrophilic Gelucires^®^ (Gelucire^®^ 50/13 and Gelucire^®^ 48/16 in different ratio) of three BCS class II model compounds (carbamazepine, CBZ, tolbutamide, TBM, and cinnarizine, CIN) having different physicochemical properties (logP, pKa, Tm) were produced by spray congealing process. The obtained MPs were investigated in terms of morphology, particles size, drug content, solid state properties, drug-carrier interactions, solubility, and dissolution performances. The solid-state characterization showed that the properties of the incorporated drug had a profound influence on the structure of the obtained solid dispersion: CBZ recrystallized in a different polymorphic form, TBM crystallinity was significantly reduced as a result of specific interactions with the carrier, while smaller crystals were observed in case of CIN. The in vitro tests suggested that the drug solubility was mainly influenced by carrier composition, while the drug dissolution behavior was affected by the API solid state in the MPs after the spray congealing process. Among the tested APIs, TBM-Gelucire dispersions showed the highest enhancement in drug dissolution as a result of the reduced drug crystallinity.

## 1. Introduction

Solid dispersion (SD) of active pharmaceutical ingredients (APIs) in hydrophilic carriers is a well-established strategy to increase the solubility and/or the dissolution rate of poorly water-soluble compounds. SD can be prepared using amorphous carriers (e.g., polyvinylpyrrolidone (PVP)), crystalline carriers (e.g., sugars), or semicrystalline carriers (e.g., polyethylene glycol (PEG)). In pharmaceutical semicrystalline SD, the hydrophilic carrier contains both crystalline and amorphous domains and the API can be amorphous, crystalline, or partially crystalline [1]. These systems have attracted extensive attention due to their complexity in terms of physical state, as both the carrier and the API can undergo crystalline phase modifications during production of the solid dispersion and/or during storage [1,2]; on one hand, the hydrophilic carrier can accelerate, slow down, or have no effect on the crystallization of the drug [3,4]; on the other hand, the drug can affect the crystallization of the carrier [2].

The majority of studies on semicrystalline dispersions employed PEG as a carrier. Multiple aspects about drug-PEG dispersions, including their microstructure [3,5], crystallinity [2,6], interactions [7,8], stability [9], and dissolution performances [10,11] have been described. Alternatively to PEG, a class of hydrophilic surface-active PEG derivatives called Gelucires^®^ has been proposed as a carrier for the production of semicrystalline solid dispersions [12]. In general, Gelucires^®^ are a family of semi-solid or waxy excipients with an amphiphilic nature, characterized by two numbers, the first one referring to the melting point and the second one referring to the Hydrophilic Lipophilic Balance (HLB) value. Starting from the early 1990s, these materials have been used as bases for modified-release dosage forms, as the wide range of hydrophobicities available allowed to obtain multiple drug-release rates, depending on the type of Gelucire^®^ chosen. Specifically, Gelucires^®^ with high HLB (44/14, 58/18, 50/13 and the most recently marketed 48/16) are composed of PEG-esters of long chain fatty acids, a small glyceride fraction and, sometimes, free PEG. Due to their structure, these carriers improved the bioavailability of poorly water soluble drugs such as piroxicam [13], meloxicam [14], and atorvastatin [15]. The mechanisms involved in the drug solubility and dissolution enhancement of Gelucire-based systems are various and include enhanced wettability due to the hydrophilic excipient, formation of micellar structures, and particle size reduction of the API [13,16]. Changes in the API solid state might also occur, but they are strictly related to the drug-to-carrier ratio and to the SD production method, i.e., by melting or solvent evaporation [14]. These and other aspects about the dissolution based-mechanisms of high HLB Gelucires^®^ (50/13 and 55/18) dispersions containing theophylline have been elucidated by Craig and coworkers [17]. In addition, the influence of the cooling rate of the melting-based process and of storage on the properties of the drug-loaded SD was highlighted [18]. Tablets based on Gelucire^®^ 50/13 solid dispersions containing caffeine and paracetamol as model drugs were also investigated upon storage. [19].

Among the different technologies useful to manufacture SD, in the last decade, the spray congealing process has attracted interest due to the advantages of being a solvent-free method, which allows to obtain free-flowing microparticles (MPs) without the need of any further step [20,21]. In a recent paper, we demonstrated the efficacy of solid dispersions prepared with high HLB Gelucires^®^ (50/13 and 48/16) by spray congealing technology in improving the oral bioavailability of the Biopharmaceutic Classification System (BCS) class II drug indomethacin [22]. Specifically, the recently marketed Gelucire^®^ 48/16 [23] demonstrated excellent properties in enhancing the solubility and dissolution rate of indomethacin, as well as its bioavailability in vivo. The bioavailability enhancement was mainly attributed to the loss of drug crystallinity when formulated into Gelucire-based solid dispersions [22]. These observations underline the importance of the solid-state behavior of drug-Gelucire dispersions as the main factor influencing the biopharmaceutical properties of the system. However, less work has been performed on the effects of the incorporation of drugs with different characteristics to the structure of the final systems and there is a limited understanding of how drug-carrier physical states influence the overall performance of Gelucires-based dispersions produced by spray congealing. Moreover, the literature on solid dispersions containing the most recently marketed Gelucire^®^ 48/16 is still extremely restricted. 

**Figure 1:**
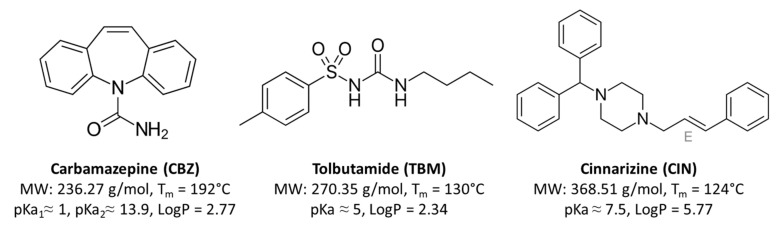
Molecular structures of model compounds and key physicochemical parameters.

Therefore, the aim of this research is to investigate the influence of the API properties on the characteristics of semi-crystalline solid dispersion based on Gelucire^®^ 50/13 and Gelucire^®^ 48/16 prepared by spray congealing. Three BCS class II APIs (Figure 1) have been selected, having different physicochemical parameters. Specifically, carbamazepine (CBZ), tolbutamide (TBM), and cinnarizine (CIN) were selected as neutral, acid, and basic compounds, respectively. The systems containing the model drugs were studied in terms of solid-state properties using various techniques (spectroscopic, microscopic, and thermal ones) in order to investigate possible solid state modifications and interactions between drugs and carrier resulting from the melting-based production process. In addition, the solubility and dissolution performances of the drug loaded-MPs were evaluated. Further, the possible correlations between physicochemical properties of the APIs, their physical state in solid dispersion, and in vitro performance were discussed.

## 2. Materials and Methods

### 2.1. Materials

APIs (CBZ, TBM and CIN) were purchased from Sigma-Aldrich (Steinheim, Germany) and used “as received”. Gelucire^®^ 50/13 and Gelucire^®^ 48/16 were kindly donated by Gattefossè (Milan, Italy). All other chemicals were of analytical grade.

### 2.2. Preparation of Samples

MPs were produced by spray-congealing technology using the Wide Pneumatic Nozzle (WPN) atomizer. Initially, the excipient (Gelucire^®^ 50/13 and Gelucire^®^ 48/16 in different ratio) was heated up to a temperature of 5 °C above its melting point. The API (10% *w*/*w*) was added to the molten carrier and magnetically stirred to obtain a suspension, which was loaded into the feeding tank. The temperature of the nozzle and the inlet air pressure were set at 55 °C and 3 bar, respectively. The atomized molten droplets hardened during the fall into a cylindrical cooling chamber, which was held at room temperature. Finally, the MPs were collected from the bottom of the cooling chamber and stored in polyethylene closed bottles at 25 °C. Three different formulations were produced for drug-loaded MPs (Table 1). For comparison purposes, unloaded MPs and physical mixes (Phy mix) of API and excipients in the same weight ratio as the loaded MPs (10% *w*/*w* of API with 90% *w*/*w* of unloaded MPs) were prepared. All analyses were performed on freshly prepared MPs.

### 2.3. Morphological Analysis of MPs and Drug Content Determination

#### 2.3.1. Morphological Analysis

Shape and surface morphology of APIs and MPs were observed by Scanning Electron Microscopy (SEM). Samples were fixed on the sample holder with double-sided adhesive tape, sputter coated with Au/Pd under argon atmosphere performed using a vacuum evaporator (Edwards, Crawley UK) and examined by means of a scanning electron microscope SEM (Philips XL 30) operating at 20.0 kV accelerating voltage. The MPs size distribution was evaluated by sieve analysis, using a vibrating shaker (Octagon Digital, Endecotts, London, UK) and standard sieves (Scientific Instruments, Milan, Italy) of 75, 150, 250, 355, and 500 μm.

#### 2.3.2. Analytical Methods

CBZ and TBM were detected by measuring their absorbance with a Cary 60 UV-Vis spectrometer (Agilent Technologies GmbH, Waldbronn, Germany). A reverse phase HPLC method was used for the quantification of CIN, due to its very limited water solubility. The HPLC system consisted of two mobile phase delivery pumps (LC-10ADvp, Shimadzu, Japan), a UV–Vis detector (SPD-10Avp, Shimadzu, Japan), and an autosampler (SIL-20 A, Shimadzu, Japan). A Luna C18 column (150 mm × 4.60 mm, 5 µm) was used with a mobile phase consisting of 45% of water acidified with 0.1% trifluoroacetic acid and 55% of acetonitrile. The injection volume was 20 µl, the flow rate was 1 mL/min, and the detection wavelength were set at 251 nm. Standards (0.1–50 µg/mL) were prepared in acetonitrile and diluted in mobile phase, where the curve exhibited good linearity (R^2^ = 0.999).

#### 2.3.3. Determination of Drug Content

Drug loading was determined by dissolving or melting a variable quantity of MPs accurately weighed in a suitable solvent. For CBZ, MPs were added to 100 mL of distilled water. The sample was heated to 60 °C to melt the carrier and then shaken for 24 h at 25 °C. Finally, the solution was filtered, diluted with water, and the drug content was assayed spectrophotometrically at 285 nm (linearity curve in the range of 0.5–50 mg/L, R^2^ = 0.999). TBM content was determined by dissolving MPs in 10 mL of ethanol. The solution was shaken for 24 h at 25 °C. After filtration and dilution with the same solvent, the drug content was assayed spectrophotometrically at 229 nm (linearity curve in the range of 5–30 mg/L, R^2^ = 0.999). CIN content was determined by adding MPs in 10 mL of acidic water, heating to 60 °C to melt the carrier, and then shaken for 24 h at 25 °C. The solution was filtered, opportunely diluted with the mobile phase, and assayed by HPLC. Each formulation was analyzed in triplicate and the mean ± SD was reported. The drug content (%) was calculated following Equation (1):

Drug content (%) = (drug (mg))/((drug + carrier)(mg)) × 100(1)

### 2.4. Solid State Characterization of MPs

#### 2.4.1. Differential Scanning Calorimetry (DSC) Studies

DSC analysis were performed using a Perkin Elmer DSC 6 (Perkin Elmer, Beaconsfield, UK) with nitrogen as purge gas (20 mL/min). The instrument was calibrated with indium and lead for temperature, and with indium for the measurements of the enthalpy. Samples of APIs, unloaded MPs, and loaded MPs, weighing 8–9 mg, were placed in an aluminum pan and heated from 25 to 220 °C at a scanning rate of 10 °C/min.

#### 2.4.2. Fourier Transform-Infrared Spectra (FT-IR) Analysis

Studies of infrared spectra of pure drugs, unloaded MPs, and loaded MPs were conducted with an IR spectrophotometer (Jasco FT-IR A-200, Milan, Italy) using the KBr disc method. The samples were mixed with KBr and compressed into tablet (10 mm in diameter and 1 mm in thickness) using a manual hydraulic tablet presser (Perkin Elmer, Norwalk, CT, USA) at 4000 kg/cm for 4–6 min. 

#### 2.4.3. Hot Stage Microscopy (HSM) Analysis

Physical changes in the samples during heating were monitored by HSM studies using a hot stage apparatus (Mettler-Toledo S.p.A., Novate Milanese, Italy) mounted on Nikon Eclipse E400 optical microscope connected to a Nikon Digital Net Camera DN100 for the image acquisition. The samples were equilibrated 25 °C for 1 min, heated at a scanning rate of 10 °C/min in the desired ranges of temperature, and cooled down to 25 °C using the same scan speed. The magnification was set at 10×.

#### 2.4.4. Powder X-Ray Diffraction (PXRD) Analysis

Single components, MPs, and corresponding physical mixtures were studied by X-ray powder diffraction technique using an X’Pert powder diffractometer (Malvern Panalytical, Almelo, NL, USA) equipped with a graphite monochromator in the diffracted beam. Cu Kα radiation was used (40 mA, 40 kV) and the spectra was obtained in the range 3°–35° 2θ.

### 2.5. Solubility and Dissolution Tests of MPs

The solubility and dissolution tests have been performed in different media depending on the properties of the API: CBZ was tested using purified water because its solubility is independent of the pH of the medium [24,25]. In case of the acid and basic model APIs, the medium has been selected in order to simulate the least favorable (and more challenging) pH condition and to better evaluate the effect of the formulation. Accordingly, TBM was tested in gastric simulated conditions (HCl solution pH 1.2) and CIN in intestinal simulated conditions (phosphate buffer solution pH 6.8).

#### 2.5.1. Solubility Studies

Solubility measurements at equilibrium of APIs, loaded MPs, and Phy mix were performed at 25 °C. An excess of the sample was added to 10 mL of the respective medium (purified water, solution at pH 1.2 and buffer solution at pH 6.8 for the CBZ, TBM, and CIN samples, respectively). The samples were magnetically stirred for 48 h, equilibrated for 2 h, and the suspensions were then centrifuged at 10.000 rpm for 10 min. The supernatant was filtered through a 0.20 μm membrane filter. For CBZ and TBM, the filtrates were suitably diluted with the same solvents and analyzed by UV-Visible spectrophotometer at 285 nm (linearity curve in the range of 0.5–50 mg/L, R^2^ = 0.999) and 231 nm (linearity curve in the range of 0.5–20 mg/L, R^2^ = 0.999), respectively. For CIN, after suitable dilution, the samples were analyzed by HPLC. Each sample was analyzed at least in triplicate.

#### 2.5.2. Dissolution Studies

The USP paddle apparatus (Pharmatest, Steinheim, Germany) was used with a rotating speed of 50 rpm. For CBZ and TBM, samples of pure drug, physical mixes, or loaded MPs (size fraction 150–250 μm) were added to 500 mL of the respective dissolution medium maintained at a temperature of 37 °C. The aqueous solution was filtered and continuously pumped (12.5 mL/min) to a flow cell in a Cary 60 UV–Vis spectrometer (Agilent Technologies GmbH, Waldbronn, Germany) and absorbance values were recorded at 285 nm and 231 nm for CBZ and TMB, respectively. In case of CIN, samples of pure drug and loaded MPs (size fraction 150–250 μm) were added to 1 L of phosphate buffer solution pH 6.8 at 37 °C. At specific time points, 2 mL of the medium were withdrawn using an 8 µm filter to avoid the removal of the MPs and the amount of drug was quantified by HPLC. Two mL of fresh medium were added to keep the volume constant. The amount of solid dispersion (and physical mixtures) was 80-100 mg for samples containing CBZ and TBM, whereas 10–15 mg for samples containing CIN. The dissolution tests were performed on six samples.

### 2.6. Statistical Analysis

One-way analysis of variance (ANOVA) followed by the Bonferroni posthoc test (GraphPadPrism, GraphPad software, Inc., San Diego, CA, USA) was used.

## 3. Results and Discussion

A range of hydrophobic APIs (Figure 1) with different properties was selected to prepare semicrystalline SD using two different high HLB Gelucires, the widely employed Gelucire^®^ 50/13 and the most recent and less investigated Gelucire^®^ 48/16. With the purpose to compare Gelucire^®^ 50/13 and Gelucire^®^48/16 as carriers for solid dispersions of poorly water soluble drugs, three formulations having different Gelucires ratios were produced: one containing only Gelucire^®^ 50/13 (formulation A), one containing both carriers in equal weight ratio (formulation B), and one containing majority of Gelucire^®^ 48/16 (formulation C). Preliminary spray congealing experiments showed that it was not possible to obtain solid MPs based entirely on Gelucire^®^ 48/16; in fact, after atomization, the droplets could not harden during the fall into the cooling chamber, kept at room temperature. Therefore, the formulation C was produced with the highest possible amount of Gelucire^®^ 48/16, equal to 70% *w*/*w*.

The characteristics of the SD obtained by spray congealing technology are discussed below. 

### 3.1. Morphology and Drug Content of the Microparticles

The composition of the MPs having a theoretical drug loading of 10% *w*/*w* are reported in Table 1. All formulations were successfully processed by spray congealing resulting in spherical and non-aggregated MPs with quite smooth surface.

**Figure 2:**
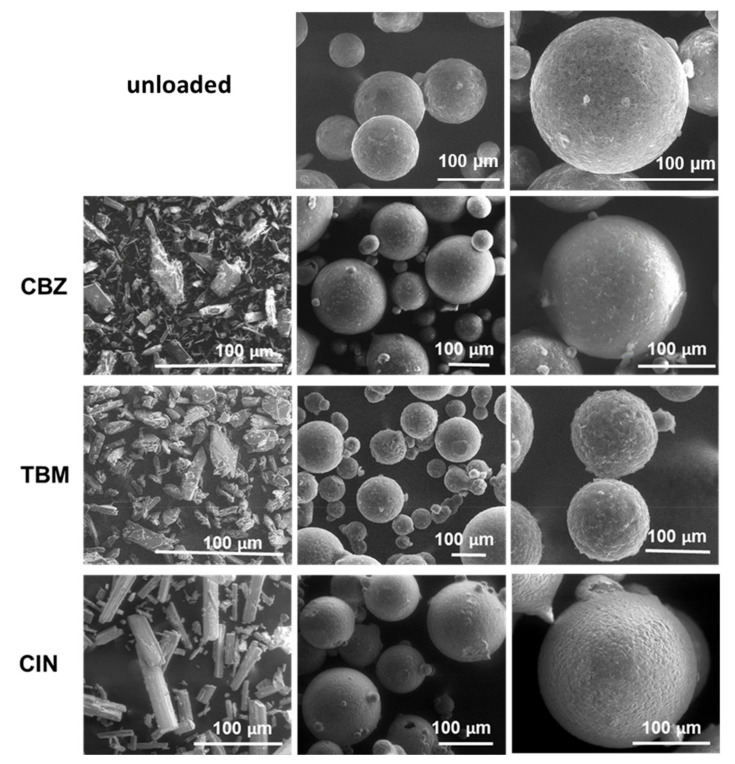
SEM images of unloaded MPs C and MPs C loaded with the model APIs at two different magnifications (center and right). The pure APIs are also shown (left).

**Figure 3:**
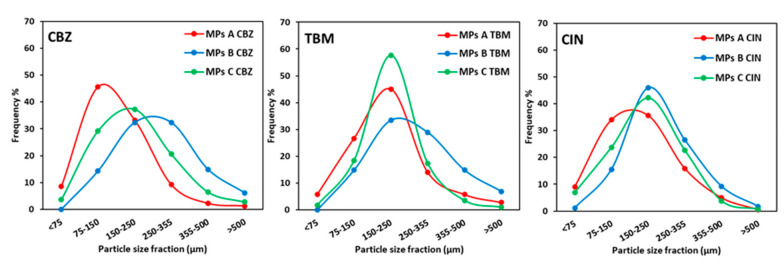
Particle size analysis of MPs A, B, and C loaded with the three model APIs.

Figure 2 reports as example the SEM images of MPs C containing the different APIs compared with the drug-free (unloaded) MPs C. The appearance of the API-loaded MPs, including shape and sphericity, was similar to the unloaded ones. The particle size analysis of the MPs (Figure 3) showed a Gaussian size distribution from 75 to 500 μm. In general, the prevalent particle size fraction was in the range of 150–250 μm. However, MPs A tended to have slightly smaller size compared to MPs B and C (e.g., MPs A CBZ main particle size fraction was 75–150 μm), while formulation B showed a slightly broader size distribution with a shift to bigger dimensions (as seen for CBZ and TBM) compared to MPs A and C. Thus, the particle morphology and size appeared to be more influenced by the carrier composition with respect to the type of API loaded.

For all model APIs, the actual drug content (Table 1) was similar to the theoretical drug content used in the preparation of the particles (10% *w*/*w*); hence, the encapsulation efficiency was higher than 80%. This is due to the spray congealing technology that ensures high efficiency of encapsulation without loss of drug during process [21]. Slightly lower drug content values were observed for CIN compared to the other two APIs. The reason might be in the particle size of the commercial API. For CIN, raw drug powder had a larger particle size compared to the other two APIs (Figure 2), resulting in a slight decrease in drug loading.

### 3.2. Solid State Characterization of the MPs

As a result of the spray congealing process, the components of the SD may undergo modifications of their physical states. Thus, it is essential to investigate the solid-state properties of the spray congealed systems. Specifically, different techniques including spectroscopic, microscopic, and thermal ones were employed to investigate possible drug solid-state modifications and interactions between drugs and carrier.

#### 3.2.1. Characterization of MPs containing CBZ

For CBZ, at least four polymorphs and a dehydrate form have been described in the literature. Table 2 reported the properties of the five forms, adopting nomenclature used by Grzesiak et al. [26].

**Figure 4:**
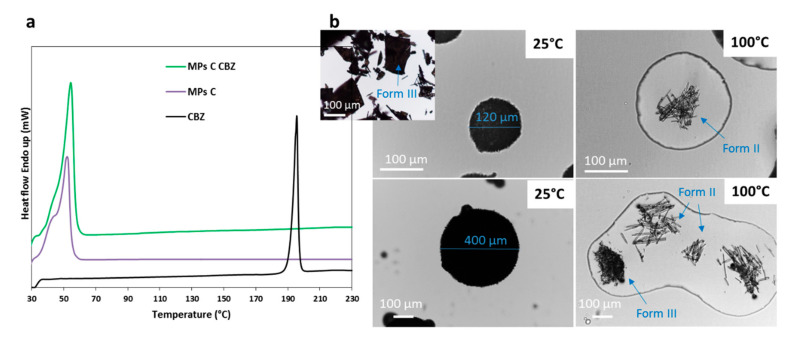
(**a**) DSC curve of CBZ, unloaded MPs C and MPs C CBZ; (**b**) HSM analysis of CBZ (small image on the left at 25 °C) and MPs C CBZ at 25° and 100 °C of small size (on the top) and large size (bottom).

First, thermal investigation of MPs was performed by means of DSC and HSM. DSC thermograms of CBZ, MPs C CBZ, and unloaded MPs C are reported in Figure 4a. CBZ melted at 192 °C with an enthalpy (*ΔH*) of fusion of 112.15 J/g. DSC curves of loaded and unloaded MPs showed only one endothermic peak at the melting temperature of the carrier in the range 33–56 °C. It is well known that, after a heating treatment, Gelucires thermal profiles are characterized by an additional lower temperature melting peak related to the solidification from the melt in a less stable solid form [19,27]. As this aspect has been already clarified in previous studies, our characterization focused on the comparison between unloaded and drug-loaded MPs rather that the effect of spray congealing process itself, in order to investigate the mutual influence of the API on Gelucire and vice versa. The *ΔH* values of MPs C (145.3 J/g) and MPs C CBZ (149.5 J/g) endotherms are similar, indicating that the presence of 10% *w*/*w* of CBZ did not affect the carrier crystallization. Notably, the absence of CBZ melting peaks might indicate the loss of drug crystalline structure after spray congealing process. The same phenomenon was observed for MPs A and B (data not shown). 

As shown by SEM (Figure 2) and HSM (Figure 4b), CBZ crystals appeared as principally plate shaped, typical of the stable polymorphic form III [28,29]. Figure 4b shows selected micrographs of HSM analysis of MPs C CBZ at 25 °C and at 100 °C. After melting of the carrier, drug crystals were observed in the molten carrier. Thus, the absence of a drug melting endotherm in the DSC thermogram of the MPs did not indicate the presence of amorphous drug in the particles, but rather the effect of a gradual drug dissolution in the melted carrier during the scan [24,30]. The slight endothermic deviation from baseline observed in the DSC curve of MPs C CBZ (Figure 4a) supported this theory [31]. Observing the HSM images, two different CBZ crystal morphologies can be noted after melting of a large particle: the original plated-shaped crystals as well as long needles-liked crystals. On further heating, both crystals gradually solubilized into Gelucire and the solubilization was complete at around 140 °C, thus below the melting temperature of the pure API. Interestingly, only long needles-like crystals were noted in smaller (< 355 µm) MPs. This observation can be explained assuming that CBZ powder partially dissolved in the molten carrier during the production process, which appeared as a suspension by visual examination. The undissolved drug fraction, with crystals up to 100–150 µm size, were mostly included into large MPs, whereas the dissolved drug fraction was included indifferently in MPs of all size. Upon cooling, the dissolved drug recrystallized in a different crystal habit, i.e., the long needles.

**Figure 5:**
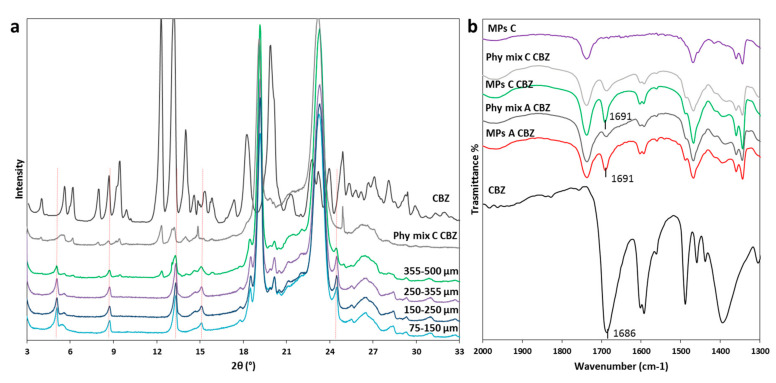
(**a**) XRPD spectra of different size fractions of MPs C CBZ compared to CBZ and Phy mix C CBZ; (**b**) FT-IR spectra of CBZ, MPs A CBZ, Phy mix A CBZ, MPs C CBZ, Phy mix C CBZ and unloaded MPs C in the spectral region 2000–1300 cm^−1^.

This hypothesis was confirmed by PXRD data, shown in Figure 5a. The XRPD pattern of pure CBZ showed diffraction peaks at 7.9, 8.6, 9.3, 12.2, 13.1, 13.9° of 2θ characteristic of form I (the stable form at high temperature), and some peaks with lower intensities at 15.2, 15.8, 17.2, 24.9 of 2θ, typical of form III. These data indicated that the starting drug was a mixture of the two stable forms of CBZ. The PXRD of Phy mix C presented the typical signals of triglycerides, observed at 19.1 and 23.3° of 2θ [32], as well as the distinctive peaks of the drug, with decreased intensity due to the low content of CBZ in the samples. Moving to the MPs, the diffractogram of MPs C CBZ showed a completely different pattern with respect to the corresponding Phy mix: beside the two intense peaks of the carrier, which showed no modification, the drug displayed new diffraction peaks at 5.1, 8.7, 13.3, 15.1, 18.5, 20.1 and 24.5°, indicated by vertical lines. Comparing these values of 2θ positions with the diagnostic peaks of the four polymorphs of CBZ, a perfect correspondence with CBZ form II was found. Interestingly, the comparison of PXRD data of different MPs size fractions confirmed our hypothesis: drug original solid form was partially maintained in large MPs, indicating a preferential encapsulation/inclusion of crystals into big particles. The metastable polymorph II was observed in all samples, indicating a certain degree of drug solubilization in the carrier during the process, followed by crystallization into a new metastable polymorph, i.e., form II. The anhydrous form II of CBZ is considered an impurity of CBZ Form III [28] and its morphology corresponds to long needles [33]. Thus, these findings were supported by the results previously obtained by HSM, which showed long regular needle-shaped crystals inside MPs C. The same result was obtained for MPs A and B (data not shown). This particular change in the CBZ solid state has been previously noticed in dispersion of CBZ in Gelucire^®^ 50/13 prepared either by melting [34] or solvent evaporation [35], as well as in PEG-based CBZ dispersions [31,36].

Infrared spectroscopy may reveal additional information about changes in API solid state and/or interactions with the carrier upon MPs production. As the carbonyl amide group of CBZ is potentially involved in interactions, the spectral region 2000–1300 cm^−1^ was examined (Figure 5b). The IR spectra of the CBZ-loaded MPs were similar to those of the Phy mix and showed all the CBZ and carrier characteristics bands, indicating the absence of drug-carrier interactions. However, for the MPs containing CBZ, the characteristic signal at 1686 cm^−1^, due to the carbonyl stretching vibration [37], was moved at 1691 cm^−1^. This band shift was evident for all formulation of MPs (data not shown for MPs B CBZ), but not in their corresponding Phy mix, in which the band remained at the original position at 1686 cm^−1^. In crystalline CBZ, the carboxyl groups are not free but involved in intermolecular hydrogen-bonding, determining a specific packing and orientation of the molecules, which are different in the various crystal forms. Thus, the shift of the carbonyl stretching band can be attributed to the hydrogen bonding rearrangement resulting from the conversion into polymorph II. Accordingly, the main infrared bands of CBZ tend to shift to higher wavenumbers passing from the stable polymorphic forms (I and III) to the trigonal form II [38] and, specifically, the carbonyl stretching band of the trigonal form II has been reported at 1690 cm^−1^ [26] and 1691cm^−1^ [38].

The overall data indicated that most CBZ solubilized in the molten carrier composed of Gelucires^®^ 50/13 and 58/16 at different weight ratio during spray congealing process and this drug fraction recrystallized in a different polymorphic form (form II) upon cooling.

#### 3.2.2. Characterization of MPs Containing TBM

Same as CBZ, TBM also exists in different polymorphic forms, schematized in Table 3.

**Figure 6:**
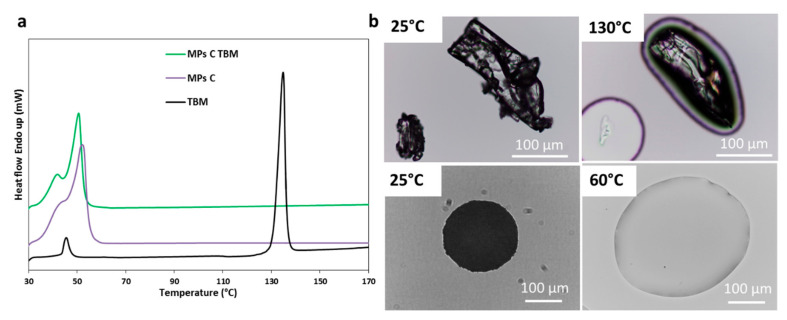
(**a**) DSC of TBM, unloaded MPs C, and MPs C TBM; (**b**) HSM images of TBM (on the top) and MPs C TBM (on the bottom).

Figure 6a showed the DSC curves of TBM, unloaded MPs C, and MPs C TMB. DSC analysis of raw drug shows a transition peak at T = 44.8 °C with an enthalpy of melting of *ΔH* = 9.5 J/g. According to the literature, this transition peak is due to the transition of form I^L^ to I^H^ [39,40]. The second endothermic event at T = 130.0 °C (*ΔH* = 98.1 J/g) is due to form I^H^ melting, in accordance with previous data [41]. Thus, DSC results of the raw material suggested that the drug was mainly in its most stable form I^L^. The melting of the carrier is represented by an endothermic event between 35 and 55 °C. However, the endothermic peak of MPs containing the drug is at temperatures slightly lower (50 °C) than that of the pure carrier (52 °C). This observation indicates a certain degree of miscibility between the drug and molten carrier and could be attributed to the formation of an eutectic mixture. The formation of eutectics has been noted for small amounts of drugs with another semicrystalline carrier, PEG [31,42,43]. It should be noted that the endothermic signal of TBM melting in the drug loaded-MPs C was absent, similarly to the previous model drug. 

HSM analysis (Figure 6b) of TBM showed prismatic crystals, which melted at 130 °C. TBM-loaded MPs C melted between 45 and 60 °C and, differently from MPs containing CBZ, no crystal was observed into the Gelucire molten matrix. The absence of drug crystals suggested the transformation of the drug into the amorphous form with loss of crystal structure. Otherwise, it is possible that drug crystals, although present in the solid particles, solubilized together with the carrier, or really close to it, consistently with the hypothesis of partial drug solubility in the carrier with the formation of an eutectic mixture.

**Figure 7:**
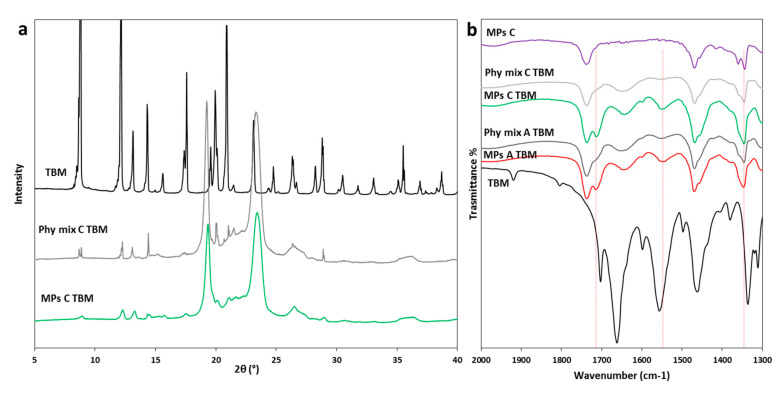
(**a**) XRPD spectra of TBM, Phy mix C TBM, and MPs C TBM; (**b**) FT-IR spectra of TBM, MPs A TBM, Phy mix A TBM, MPs C TBM, Phy mix C TBM and unloaded MPs C in the spectral region 2000–1300 cm^−1^.

XRPD analysis was performed to further investigate the solid state of TBM into MPs after the spray congealing process, using formulation C as an example. As shown in Figure 7a, pure TBM is highly crystalline with diffraction peaks at 8.8, 12.2, 13.2, 14.3, 17.6 and 20.9 of 2θ, typical of form I^L^ [39,44]. The diffractogram of Phy mix C TBM showed sharp peaks at the same angle of 2θ, with lower intensity due to the amount of TBM (10% *w*/*w*) in the sample. When the TBM was loaded in MPs C, the diffraction peaks of the drug appeared slightly broader and with lower intensity with respect to the Phy mix. This result excluded the conversion into a different polymorph and could indicate the partial conversion in the amorphous form and/or the formation of TBM crystals with smaller size (e.g., nano or micro crystals). Thus, this result was consistent with the DSC and HSM findings and confirmed the presence of a certain degree of drug solubility in the molten Gelucire, resulting in lower residual drug crystallinity in the MPs C. Similar results were obtained for MPs A and B (data not shown). The possibility of the formation of a molecular dispersion of TBM using PEG as carrier as a result of drug solubilization in molten carrier has been previously suggested [45,46].

The FT-IR analysis of TBM samples is reported in Figure 7b. The most significant changes in the IR spectra of MPs with respect to the spectrum of raw TBM are in the 2000–1300 cm^−1^ region. In the spectra of TBM-loaded MPs A and C, a band appeared at 1714 cm^−1^. This band could be attributed to the carbonyl stretching of the drug when the C=O group is in a non-polar environment, as previously reported for TBM comelt with PEG [45]. Moreover, the strong band at 1661 cm^−1^ due to TBM carbonyl stretching vibration appeared as a large band at ca. 1641cm^−1^ in the spectra of TBM-loaded MPs and Phy mix. The band at 1555 cm^−1^, related to the NH bending (amide II), also became broader and shifted to lower wavenumbers: 1545 cm^−1^ and 1551 cm^−1^ for TBM-loaded MPs and Phy mix, respectively. These changes involved not only signals attributed to the API, but also bands of the carrier (e.g., the double band of the carrier at 1344–1359 cm^−1^ due to C–C stretching vibration of Gelucire is replaced by a single strong band at 1346 cm^−1^), confirming the presence of a mutual interaction drug-carrier. Specifically, hydrogen bonding between the drug and carrier would reduce the number of intermolecular hydrogen bonds of the drug itself; hence, the observed shift to a higher wave number is indicative of less hydrogen bonding for the API functional groups. These changes, more evident for MPs compared to the corresponding Phy mix, suggested a partial displacement of the original arrangement of CBZ molecules in the I^L^ crystals and the establishment of interactions with the carrier as a result of incorporation into the MPs.

#### 3.2.3. Characterization of MPs Containing CIN

The basic drug CIN is, among the three model APIs, the most lipophilic compound with a logP of 5.8 [47]. It is, therefore, reasonable to expect lower affinity between the drug and the hydrophilic carrier Gelucire. Regarding the solid state of CIN, literature data reported only one crystalline form. 

**Figure 8:**
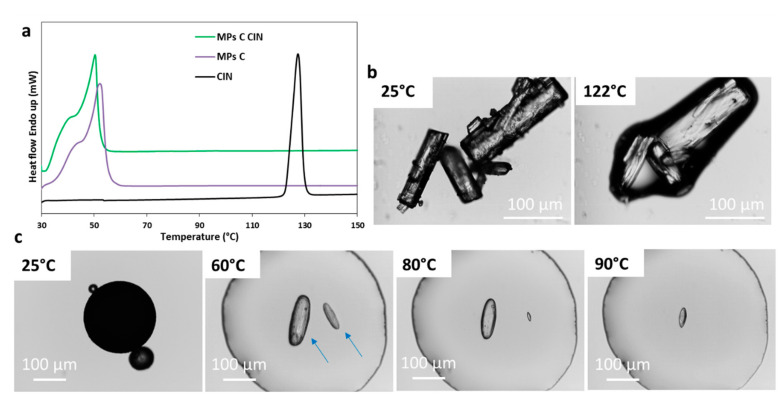
(**a**) DSC of CIN, unloaded MPs C, and MPs C CIN; (**b**) HSM images of CIN; (**c**) HSM images of MPs C CIN.

The DSC data are showed in Figure 8a. The melting of the drug occurred at 122–124 °C. Similar to the other APIs, the drug melting endotherm was absent in the curve of CIN-loaded MPs C. As observed for TBM, in the case of CIN as well, the endothermic peak of Gelucire in the drug-loaded MPs was shifted to slightly lower temperatures (50 °C) compared to the pure carrier (52 °C), suggesting a certain degree of API solubility in the molten carrier. As shown in Figure 8b, CIN original crystals are regular column-shaped crystals, which melted at 122 °C in agreement with the endothermic event observed by DSC. The HSM analysis of MPs (Figure 8c), showed that crystals of CIN can be observed upon carrier melting (arrows in Figure 8c). Specifically, the crystals had similar morphology to the original one, but with smoother rounded borders. The API progressively solubilized in the carrier with the increasing of the temperature, and the solubilization was complete within 100 °C. Again, this observation explained the lack of drug endothermic peak in the DSC and confirmed the property of molten Gelucire in solubilizing the API crystals in a temperature-dependent manner.

**Figure 9:**
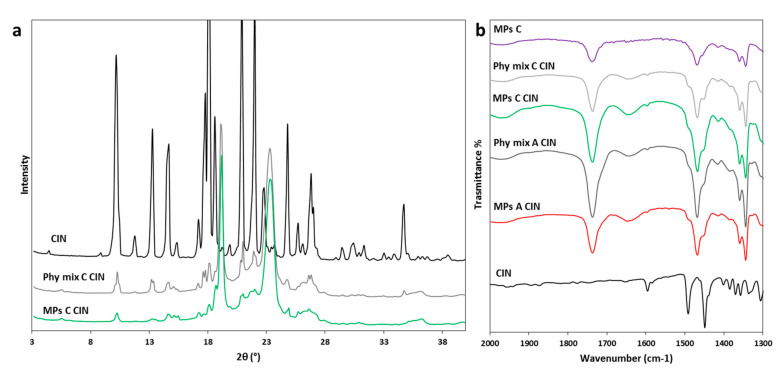
(**a**) XRPD spectra of CIN, Phy mix C CIN and MPs C CIN; (**b**) FT-IR spectra of CIN, MPs A CIN, Phy mix A CIN, MPs C CIN, Phy mix C CIN and unloaded MPs C in the spectral region 2000–1300 cm^−1^.

Figure 9a displays the PXRD data of CIN samples. CIN was highly crystalline with diffraction peaks at 10.2, 13.3, 17.7, 20.9, 22.8, 24.8, and 34.7°of 2θ. Diffraction peaks at the same position 2θ were observed for both MPs CIN C and corresponding Phy mix. The PXRD pattern of MPs showed broader and less intense reflections related to the API, compared to the physical mixture one. This observation is consistent with the presence of smaller drug crystals in the MPs noted in HSM analysis (Figure 8c) as a result of a partial drug solubilization during the spray congealing process. Similar results were obtained for MPs A and B (data not shown). For CIN, the FT-IR analysis (Figure 9b) did not show any evidence of drug-carrier interaction, as the main bands of the carrier are in the same positions for MPs A and C and for the corresponding Phy mix.

Therefore, the solid-state characterization of the drug-Gelucire SD produced by spray congealing process showed a different behavior for each API. Table 4 summarizes, for each model compound, the main information revealed by the various analytical techniques employed and the overall conclusion about the API physical form in the solid dispersion. 

Given the complexity of the spray congealed systems, the correlation between physicochemical properties of the APIs and their behavior in the dispersion is not straightforward. The first step of the spray congealing process consists of the addition of the API to the molten carrier; as long as the carrier is in the molten state, it acts as a solvent and the API acts as a solute. Our results indicated that for all model APIs, the amount of solubilized drug in the molten Gelucire was limited, as the amount of drug added (10% *w*/*w*) was not entirely solubilized. In this regard, the lipophilicity and polarity of the drug play an important role. In the context of lipid-based formulations, it has been suggested that APIs with intermediate logP (between 2 and 4) present higher solubility in lipid formulation containing hydrophilic surfactants, while APIs with logP > 4 would achieve adequate solubility in highly lipophilic vehicles (e.g., pure triglycerides) [48]. Thus, from this point of view, the solubilization of CBZ and TBM (2 < logP < 3) in molten Gelucire would be expected at a higher extent compared to CIN (logP > 5). 

The last step of the process involves the cooling of the atomized fluid; in this phase, both the (solubilized) API and the molten carrier solidify. Interestingly, the solubilized drug fractions behaved differently: HSM analysis showed that CBZ re-crystallize upon cooling, whereas no evident crystallization was observed for TBM and CIN using the same cooling conditions. This can be explained considering the different melting temperature (T_m_) of the APIs. CBZ, having a higher melting point compared to TBM and CIN (Figure 1), is expected to have a stronger crystallization tendency. In fact, for higher melting material, the temperature difference (ΔT) between its T_m_ and the undercooled liquid is higher; thus, the driving force for nucleation and crystallization is larger, as stated by Baird et.al in a previous work on the crystallization tendency of organic molecules from their melts [49]. Specifically, ΔT was 127 °C for CBZ, 65 °C for TBM and 59 °C for CIN. There is literature evidence of the high crystallization tendency of CBZ, both from the undercooled melt [49] and from aqueous environment [50]. In addition, for CIN, our results were consistent with the crystallization behavior reported in the literature for this drug, classified as having intermediate/slow crystallization tendency [49,50]. Although TBM was observed to crystallize easily from the melt [49] and from organic solvents [51], the formation of interactions between TBM and carrier (Figure 7b) can explain the lack of drug crystallization observed in the present study. The interaction between API and Gelucires, based on dipole interactions or hydrogen bonding, is facilitated in case of compounds having a hydrogen donor or acceptor group and with modest lipophilicity, as in the case of TBM (lowest logP value among the tested APIs). The solid-state behavior of drug/Gelucire dispersions is complex as different parameters, including the API T_m_, logP, polarity, and presence of interacting chemical groups, contribute to determining drug behavior in the solid dispersion.

### 3.3. Solubility and Dissolution Studies

**Figure 10:**
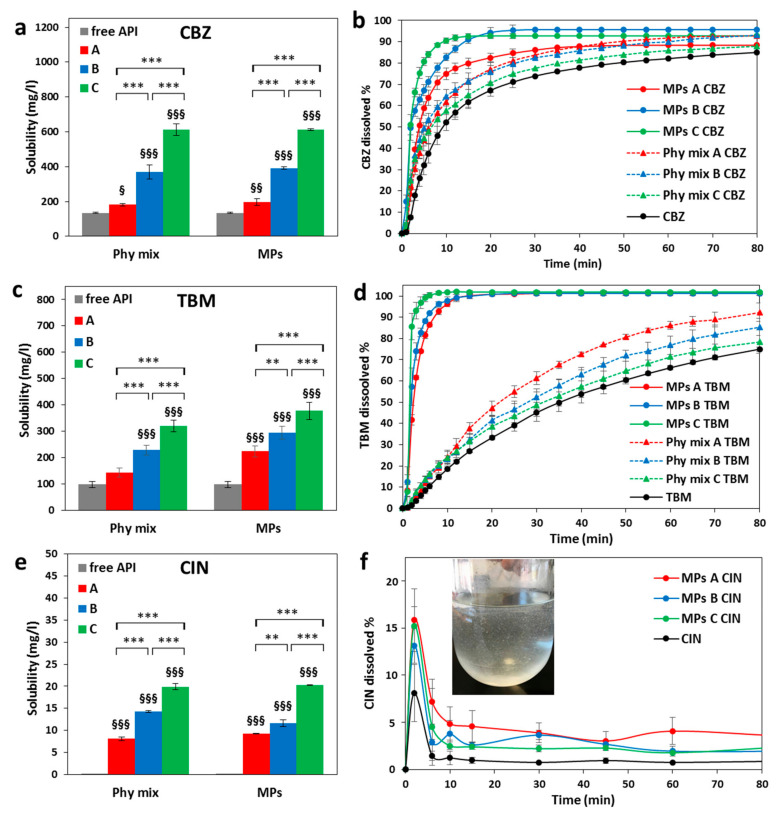
(**a**,**c**,**e**) Solubility studies of MPs and Phy mix compared to the pure APIs. Data represent mean ± S.D. (*n* = 3), the level of significance was set at the probabilities of § *p* < 0.05, §§ *p* < 0.01, §§§ *p* < 0.001 compared to the free API, and ** *p* < 0.01, *** *p* < 0.001 the statistical significance between the different formulations of Phy mix and MPs; (**b**,**d**,**f**) Dissolution profiles and of MPs A, MPs B, and MPs C compared to the pure APIs. For CBZ and TBM, a comparison of MPs dissolution profiles with the corresponding Phy mix has also been performed (dotted curves). For CIN, a photograph of the dissolution medium at the end of MPs C CIN dissolution test has been added.

Solubility and dissolution studies of the different MPs formulations containing the three drugs were performed in challenging dissolution media: purified water, solution at pH 1.2, and buffer solution at pH 6.8 for CBZ, TBM, and CIN samples, respectively, according to their neutral, acid, and base character. The results of solubility and dissolution studies are shown in Figure 10. The solubility of the APIs (Figure 10 a, c, e) was enhanced in the order MPs C > MPs B > MPs A for all model drugs. In particular, the MPs having the highest amount of Gelucire^®^ 48/16 (MPs C) increased the solubility of CBZ and TMB of about 4.5 and 3.8 -fold, respectively. Notably, the values of CBZ and TBM solubility achieved by formulation C were significantly higher compared both to the pure APIs and the formulations A and B (*p* < 0.001). Free CIN showed an extremely low solubility value: the experimental value was ~0.05 mg/L, in accordance with previous data [52]. The presence of Gelucire determined a remarkable increase in the API solubility, with the best performance (about 20 mg/L) observed by MPs C and the corresponding Phy mix (Figure 10e). Interestingly, in case of CBZ and CIN samples, the MPs and their respective Phy mix displayed similar solubility values, while for the TBM samples, the MPs exhibited a slightly higher solubility compared to the simple carrier-API Phy mix. Nevertheless, the statistical difference was not significant overall (*p* > 0.01). These results suggest that the solubility improvement was not related to the API solid state in the MPs, but rather to the ability of the amphiphilic carrier to increase API solubilization as a result of the formation of a micellar dispersion [15].

On the contrary, the results of the dissolution tests (Figure 10b,d,f) evidenced a different dissolution behavior for each of the three APIs.

Figure 10b showed that an increase in dissolution rate of CBZ was achieved by simply mixing the drug and the carrier (Phy mix), as result of a better wettability of the drug in the presence of Gelucires. However, MPs displayed the highest dissolution enhancement and the best performance was achieved by MPs C owing to their greater solubility with respect to MPs A and B. Figure 10b showed that in case of MPs, up to 80% of CBZ was dissolved in water within 10 min, while the same amount of CBZ was dissolved after 40 and 50 min, for the Phy mix and the pure drug, respectively. The better performances of MPs over Phy mix can be ascribed to the modification of the drug solid state after the spray congealing process. Additionally, higher wettability and dispersibility of the drug should be also considered as reasons of dissolution increase observed for MPs, as in SD, each single drug crystal is very intimately encircled by the soluble carrier, which can readily dissolve upon contact with the aqueous media. It is important to mention that particle size of phy mix was comparable to those of MPs, albeit the particle size of the API loaded in the MPs might be different from the free (unprocessed) drug powder, as a result of the spray congealing process.

The dissolution profiles of the acid model drug TBM in pH 1.2 solution (Figure 10d) evidenced a marked dissolution enhancement in case of MPs, with MPs C showing the highest dissolution rate, similarly to CBZ. Notably, for this drug, the difference between the MPs and the corresponding physical mixtures was more pronounced. This difference can be explained considering the higher energy state of the drug loaded into the MPs as a result of the reduced drug crystallinity (Table 4). 

A different result was obtained for CIN samples (Figure 10f). Owing to the very low and highly pH-dependent CIN solubility, the dissolution rate of pure CIN in pH 6.8 phosphate buffer was extremely low. The dissolution profiles were similar for all CIN samples, although the amount of dissolved drug was different. In the first 5 min, CIN started to dissolve (up to 8% for free API), followed by an immediate decrease in drug concentration, indicating a precipitation of excess CIN solubilized at the beginning of the test. Up to 15% of CIN was dissolved in the case of MPs. The improvement of this “spring” effect was, however, not maintained longer, as the drug precipitated in loosely aggregated clusters clearly observed in the dissolution medium (picture in Figure 10). These results suggested that at unfavorable pH conditions, where the drug solubility is extremely low, the MPs favor the formation of a supersaturated solution followed by API precipitation. 

Overall, the potential of Gelucire-based MPs as a strategy for the formulation of BCS class II drugs was evident for all three model APIs showing a great increase in solubility, whereas the increase in dissolution rate was important for TBM and CBZ and less evident for CIN. The solubility improvement was strongly dependent on the carrier composition as it followed the same trend for all model APIs (formulation C > formulation B > formulation A) without significant differences between MPs and Phy mix. The carrier composition had less influence on the dissolution rate, where the physicochemical properties of the original drug molecule as well as the physical states of drug and carrier in the MPs dictated the dissolution behavior. Specifically, the parameters of lipophilicity (logP) and acid-basic character (pKa values) are of fundamental importance in this regard. On the other hand, the physical form of the API in the dispersion affects the dissolution rate. The presence of the drug in a higher energy solid state forms, such as partially amorphous or reduction of the crystal size, has the potential of increasing the dissolution rate as less energy is required to break the drug crystalline lattice during the dissolution process.

## 4. Conclusions

Solid dispersion of three different BCS class II drugs based on hydrophilic semicrystalline carriers (Gelucire^®^ 50/13 and Gelucire^®^ 48/16 in different ratio) were successfully produced by spray congealing in form of MPs. The characteristics of the loaded API appeared to have a negligible influence on particle size and morphology of the obtained systems: all MPs were spherical, non-aggregated, with prevalent particles size in the range 150–250 μm and good encapsulation efficiency. On the other hand, a combination of various analytical techniques demonstrated that the API properties strongly affected the solid state of the MPs. In fact, whereas the carrier was mostly crystalline in all dispersions, each API showed a different behavior: CBZ recrystallized in a different polymorphic form (form II), TBM showed an important decrease in crystallinity as a result of specific interactions with the carrier, and CIN presented smaller crystals. Thus, the solid-state behavior of the drug Gelucire’s MPs is complex and numerous factors such as physicochemical characteristics of the API (Tm, log P, polarity), its affinity with the carrier, and specific interactions between drug and carrier influence the solid state of the API in the dispersion. Furthermore, the physical states of the drug in Gelucire-based systems played a key role in dictating the dissolution behavior of the dispersion, concurrently with the physicochemical properties of the drug molecule (log P and pKa). In fact, a substantial enhancement in dissolution rate of MPs compared to the Phy mix was observed for TBM, which showed a reduced crystallinity in the dispersion and a strong interaction with the carrier. Differently, the enhancement of drug solubility appeared to be mainly related with carrier composition as it followed the same trend for all model APIs (formulation C, having the highest amount of Gelucire^®^ 48/16 > formulation B > formulation A, based only on Gelucire^®^ 50/13) without significant differences between MPs and Phy mix. Overall, this study evidenced the complexity of the solid-state behavior of drug-Gelucire dispersions produced by spray congealing technology and its role in influencing the performances of the system in terms of dissolution behavior. It is finally important to underline that, beside the characteristics of the API, other factors such as spray congealing process parameters (e.g., process temperature) and drug-carrier ratio might be additional important elements in determining the physical state of the system. For the investigated MPs, the process temperature was fixed just above the melting temperature of the carrier and the drug-to-carrier ratio was 1:9. Studies are in progress to further investigate these aspects. 

## Figures and Tables

**Table 1:** Composition and actual drug content of the MPs A, MPs B, and MPs C loaded with CBZ, CIN, and TBM.

Samples	Constituents (%, *w*/*w*)			Actual Drug Content (%, *w*/*w*)		
	Gelucire^®^ 50/13	Gelucire^®^ 48/16	API	CBZ	CIN	TBM
MPs A	90	-	10	10.32 ± 0.13	9.38 ± 0.76	10.72 ± 0.87
MPs B	45	45		10.14 ± 0.06	9.37 ± 0.97	9.76 ± 0.11
MPs C	27	63		9.71 ± 0.37	8.19 ± 0.94	9.76 ± 0.27

**Table 2:** Nomenclature and main properties of the four CBZ polymorphs and the dehydrate.

Nomenclature	Crystal Structure	Morphology	XRD Diagnostic Peaks (°, 2θ)	Characteristics
I	Triclinic	Fine needles	7.9, 8.6, 9.4, 12.2, 13.1, 19.9	Stable form at high temperature
II	Trigonal	Long and thick needles	8.7, 13.3, 15.0, 18.5, 20.1, 24.5	Metastable form, considered an impurity of form III
III	*P*-Monoclinic	Plate-shaped prisms	15.3, 15.9, 17.2, 19.5, 25.0	Commercial form. Stable form at room temperature, highly hygroscopic
IV	*C*-Monoclinic	Spherical plates	14.1, 17.9, 21.8, 33.1	Considered an impurity of form III, less soluble
dihydrate	-	Fine needles	8.8, 12.1, 18.8, 19.4	Formed in humid conditions, less soluble than form III

**Table 3:** Nomenclature and main properties of the TBM polymorphs.

Nomenclature	Crystal Structure	Morphology	XRD Diagnostic Peaks (°, 2θ)	Melting Point (°C), *ΔH_fus_* (kJ/mol)	Transition Point (°C)
I^H^	-	-	-	128, *23.8*	-
I^L^	Orthorhombic	Prism	8.7, 12.1, 19.9. 20.9	-	40
II	Monoclinic	Plate	10.3, 11.3, 19.6	117, *26.0*	-
III	Monoclinic	Needle	11.2, 15.4, 18.2	-	106
IV	Monoclinic	Needle	10.6, 18.0, 18.9	-	88

**Table 4:** Main findings gained by different characterization techniques and the overall conclusion on the physical state and behavior of the model APIs in Gelucire SD prepared by spray congealing.

API	DSC	HSM	PXRD	FT-IR	Conclusion
CBZ	Absence of the API melting endotherm	Drug crystals in the melted carrier (solubilization at 60 °C–140 °C)	Definite peaks at different 2θ position	No evidence of drug – carrier interaction	Partial API solubilization in the carrier and recrystallization in different polymorph
TBM	Absence of the API melting endotherm	No drug crystals in the melted carrier (60 °C)	Broader and less intense peaks	Evidence of drug-carrier interactions	Partial API solubilization in the carrier with reduced crystallinity
CIN	Absence of the API melting endotherm	Drug crystals in the melted carrier (solubilization at 60 °C–100 °C)	Broader and less intense peaks	No evidence of drug – carrier interaction	Partial API solubilization in the carrier with formation of smaller drug crystals
